# Complexity of information processing in obsessive-compulsive disorder based on fractal analysis of EEG signal

**DOI:** 10.17179/excli2020-2783

**Published:** 2021-03-15

**Authors:** Saeid Yazdi-Ravandi, Dorsa Mohammadi Arezooji, Nasrin Matinnia, Farshid Shamsaei, Mohammad Ahmadpanah, Ali Ghaleiha, Reza Khosrowabadi

**Affiliations:** 1 Behavioral Disorders and Substance Abuse Research Center, Hamadan University of Medical Sciences, Hamadan, Iran; 2 Institute for Cognitive and Brain Sciences, Shahid Beheshti University GC, Tehran, Iran; 3 Department of Nursing, College of Basic Science, Hamadan Branch, Islamic Azad University, Hamadan, Iran

**Keywords:** electroencephalography (EEG), obsessive-compulsive disorder (OCD), fractal analysis, Katz fractal dimension, complexity

## Abstract

The human brain is considered as a self-organizing system with self-similarities at various temporal and spatial scales called “fractals”. In this scale-free system, it is possible to decode the complexity of information processing using fractal behavior. For instance, the complexity of information processing in the brain can be evaluated by fractal dimensions (FDs). However, it is unclear how over-elaboration of information processing impacts the dimensionality of its fractal behavior. In this study, we hypothesized that FDs of electroencephalogram (EEG) in obsessive-compulsive disorder (OCD) should be higher than healthy controls (HCs) because of exaggeration of information processing mainly in the frontal regions. Therefore, a group of 39 OCDs (age: 34.76±8.22, 25 female, 3 left-handed) and 19 HCs (age: 31.94±8.22, 11 female, 1 left-handed) were recruited and their brain activities were recorded using a 19-channel EEG recorder in the eyes-open resting-state condition. Subsequently, fractal dimensions of the cleaned EEG data were calculated using Katz's method in a frequency band-specific manner. After the test of normality, significant changes in the OCDs as compared to the HCs were calculated using a two-sample t-test. OCDs showed higher FDs in the frontal regions in all frequency bands as compared to HCs. Although, significant increases were only observed in the beta and lower gamma bands, mainly at the high beta. Interestingly, neurophysiological findings also show association with severity of obsessive behaviors. The results demonstrate that complexity of information processing in the brain follows an intimate nature of structural and functional impairments of the brain in OCD.

## Introduction

The human organs and mainly the brain could resemble a complex nonlinear system. Therefore, nonlinear methods such as fractal analysis could also help to better understand the functionality of the brain and its related dysfunctions (Iglesias-Parro et al., 2016[23]). The fractal theory has been used to study the nature of behavioral and cognitive dysfunctions using biological data. For instance, decrease of FDs of heart-rate variations (Kojima et al., 2008[28]) and increase of fractal fluctuations of gait temporal patterns (Aybek et al., 2012[8]) have been related to depressive behavior. Also, lower FDs of mood time series in patients with affective instability have been documented using a visual analog mood scale (Woyshville et al., 1999[49]). Fractal theory has also proven to be a suitable tool to analyze EEG data in the frequency domain (Phothisonothai and Nakagawa, 2008[39]; Zappasodi et al., 2014[53]). For instance, the FDs of EEG data from bipolar patients with a manic episode are significantly augmented (Bahrami et al., 2005[10]). Higuchi FD is extensively used in EEG signal processing to measure the complexity of the signals in the time domain (Higuchi, 1988[21]). Higuchi FDs of EEG signals from people with major depressive disorders (MDD) show higher complexity as compared to HCs (Ahmadlou et al., 2012[3]). FDs of EEG data have been used to profile mental disorders. For instance, a recent study used EEG signal's Higuchi FD as a nonlinear feature to classify depressed individuals from HCs (Hosseinifard et al., 2013[22]).

Obsessive-compulsive disorder (OCD) is a psychiatric condition (with usual onset before 35 years), distinguished as the patient having particular thoughts repeatedly (termed obsessions), performing specific “rituals”, or feeling the need to execute a task over and over again (termed compulsion) as to relieve these thoughts; such as nail biting, bathing and hand washing frequently. These recurring thoughts and actions are difficult to manage or stop for longer than a short amount of time. Adult patients are usually aware that these obsessions and compulsions are not rational. At times, such aspects of OCD makes for a burdensome day to day life (Mancebo et al., 2005[34]). OCD is also linked to behavioral tics, anxiety disorders, and a greater risk of suicide (APA, 2013[7]; Angelakis et al., 2015[5]). Another meta-analysis revealed that OCD patients tend to have extensive, yet meager cognitive deficiencies; conspicuously, vis-à-vis verbal and spatial memory, decision making, fluency, and processing speed. OCD patients exhibit impairment in developing structured approaches to coding information, motor and cognitive inhibition, and set-shifting (Abramovitch et al., 2013[1]; Çetinay Aydin and Gulec Öyekcin, 2013[12]; Greisberg and McKay, 2003[19]; Keefe, 1995[25]; Kuelz et al., 2004[29]; Rao et al., 2008[42]; Shin et al., 2008[44]; Yazdi-Ravandi et al., 2018[51][52]). These findings didn't indicate any significant auditory attention deficits (Shin et al., 2013[45]). Moreover, there have been reported cases of some types of OCD having a positive influence on specific tasks, namely spatial working memory and pattern recognition (McKay et al., 2004[35]). 

The cortico-basal ganglia-thalamo-cortical loop model (CBGTC) is originated from the finding that the basal ganglia loops associated with the orbitofrontal cortex (OFC) and anterior cingulate cortex (ACC) are involved in OCD (ergo referred to as the OFC/ACC CBGTC loops) by means of neuroimaging (Alexander et al., 1986[4]; Maia et al., 2008[33]). Obsessions commence when the circuit ceases to confine implicitly processed information, causing that information to be processed in explicit processing systems, namely the dorsolateral prefrontal cortex (DLPFC) and hippocampus, and eventually leading to obsessions (Koen and Stein, 2015[27]). The circuit's failure to confine information results in more complex EEG recordings from certain channels.

While functional changes in the brain associated with OCD have been studied using different neuroimaging methods, fairly less is discovered using the EEG technique (Ortigue et al., 2009[38]). EEG recording of the brain waves can be band-pass filtered and categorized into a number of frequency ranges including delta [1-4 Hz], Theta [4-8 Hz] , Alpha [8-13 Hz], Beta [13-30 Hz], and Gamma [>30 Hz] bands, and several sub-bands including Alpha I [8-10 Hz], Alpha [10-12 Hz], Beta I [12-15 Hz], Beta II [15-18 Hz], Beta III [18-25 Hz], Beta IV [25-30 Hz] for more details. These frequency bands correspond with certain mental states; for instance, the alpha waves correspond with attentional processes and the beta waves reflect emotional and cognitive processes (Klimesch et al., 1998[26]). Relating to OCD, preceding EEG-based researches have demonstrated that there is decreased beta and increased theta activity in the frontal and frontotemporal regions of the brain in OCD patients (Bucci et al., 2004[11]; Locatelli et al., 1996[32]; Prichep et al., 1993[40]).

In this paper, it is intended to perform fractal analysis on the EEG data recorded from OCD patients and healthy controls, as to discover significant differences in various frequency bands that would explain neurophysiological data's complexity and the channel locations that this complexity is most present in. Finally, a correlation between fractal dimension which is a measure of self-similarity, and ergo complexity, of the signal and the OCDs' scores on the Yale-Brown obsessive-compulsive scale (Y-BOCS) would be studied.

## Materials and Methods

### Participants

The raw data for this work has been collected in the psychiatric ward of Farshchian (Sina) hospital of the city of Hamadan in 2016. Out of the 39 OCD patients, 25 were female (age: 34.76±8.22), who met DSM-5 criteria for obsessive-compulsive disorder. Also, a group of 19 people, 11 of which were female (age: 31.94±8.22), made up the healthy control (HC) group. All patients had taken selective serotonin reuptake inhibitors (SSRIs). All participants signed informed written consent before taking part in the study. This study has been reviewed and given approval by the local ethical committee of the Hamadan University of Medical Sciences.

Patients satisfying the following prerequisites were admitted into the study: i) diagnosis of OCD (according to the DSM-5 criteria) by a psychiatrist which is also confirmed at the end of a clinical interview, ii) scoring at least 16 on the Yale-Brown obsessive-compulsive scale (Y-BOCS) (Goodman et al., 1989[18]), iii) aging between 18 and 60. Additionally, participants with the following conditions were excluded from the study: i) any mental disorders apart from OCD, ii) a history of substance abuse or dependency, iii) any grave accompanying medical disorders or neurologic conditions, iv) history of severe head trauma, v) intellectual impairment, vi) electroconvulsive therapy (ECT) in the span of one year prior to the study, vii) physical handicap including vision, hearing or speech impairment, paralysis or amputation, viii) pregnancy or any clinical condition affecting the EEG data significantly. 

Table 1(Tab. 1) depicts statistical data on the two groups taking part in the study. As the table shows, the p-values cross the threshold of 0.05; and therefore it could be concluded that the participants' gender, right/left handedness, and age don't dramatically change between subjects, thus the effects of the aforementioned characteristics have not been taken into account in the analysis.

### EEG recording

The EEG data were recorded from the two groups of subjects, in the Farshchian (Sina) hospital of Hamadan, from 9 to 11 am. A Cadwell Easy II Amplifier along with 19 Ag/AgCl surface electrodes, namely: Fp1, Fp2, F3, F4, F7, F8, Fz, C3, C4, Cz, T3, T4, T5, T6, P3, P4, Pz, O1, and O2 (with Cz as the reference electrode) were utilized in a 10-20 formation (international system) via Electro-Caps (Electro-Cap International, Inc.). With a sampling frequency of 200 Hz, the EEG data were acquired from participants in a resting state with open eyes. The electrode impedance was less than 5 kΩ for the duration of the experiment. 

**Figure 1:**
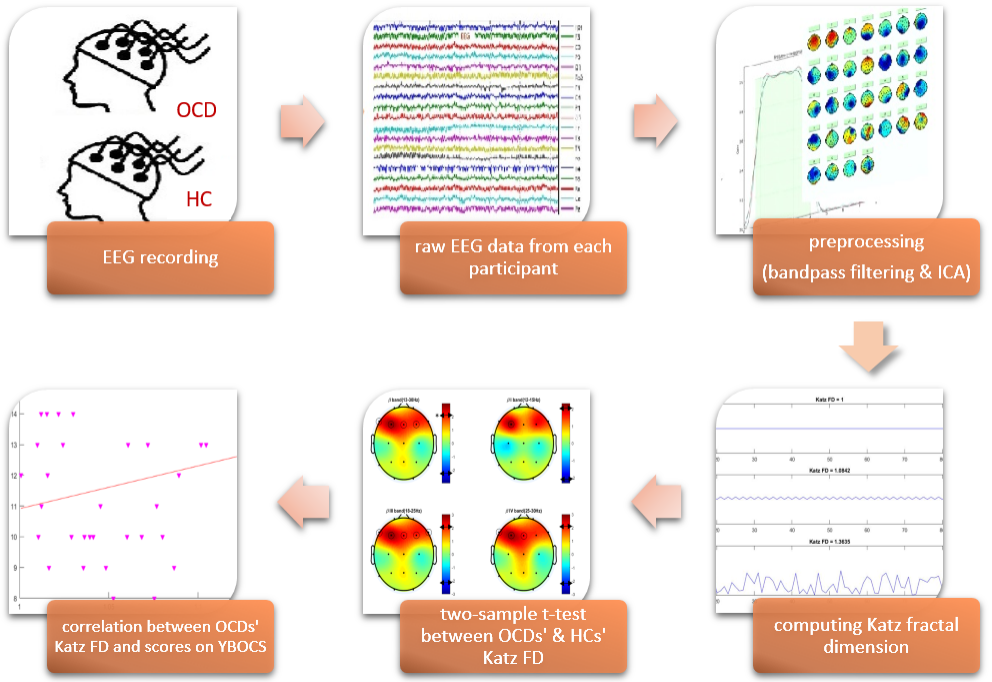
Experimental design

Figure1(Fig. 1) illustrates the experimental design. The study steps are described in the following sections.

### Preprocessing

Using a simple FIR filter with zero phase shift, the EEG data were bandpass filtered between 1 to 40 Hz. After segmenting into 3-second trials, artifacts were removed from the data, using the ICA technique, followed by visual inspection. Bad channels, according to the kurtosis method, were interpolated. Finally, EEG data were re-referenced to average. EEG data processing was performed using MATLAB R2016b (The MathWorks Inc., Natick, USA) and the EEGLAB v14.1.2b toolbox.

### Katz fractal dimension

A fractal is a natural or mathematical set that showcases a recurrent pattern in every increasingly small scale (Verma, 2016[47]). Fractal dimension calculates the effective number of degrees of freedom in a dynamical system (Clark, 1990[14]) and is a ratio that represents a statistical index of complexity (Falconer, 2003[15]). There are several approaches to compute the fractal dimension of EEG time series, including Higuchi's method (Higuchi, 1988[21]), Katz's method (Katz, 1988[24]), box counting (Liebovitch and Toth, 1989[31]). In this study, Katz fractal dimension is computed and used for fractal analysis. Katz FD is computed from:

*FD* = log(n)/(log(n)+log(*d*/*L*))

(Katz, 1988[24])

where “n” is the number of steps in the waveform (one less than the number of data points), “d” is the planar extent (diameter) of the waveform, and “L” is the total length of the waveform. With this formulation, Katz FD ranges from 1.0 for straight lines, to about 1.15 for random-walk waveforms, and 1.5 for the most convoluted waveforms (Katz, 1988[24]). 

**Figure 2:**
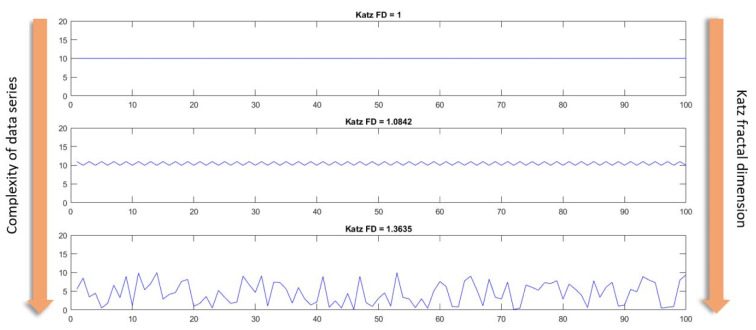
Katz FD and complexity of three different data series

Figure 2(Fig. 2) shows three different data series, arranged from simplest to most complex. The first data series is a simple horizontal straight line, whose Katz FD is equal to one. Next, the Katz FD of a periodic linear data series, which is marginally more complex than the first, is calculated to be 1.0842. Finally, the Katz FD of a completely random data series is equal to 1.3635. It can be deduced that as the complexity of a given data series increases, so does the Katz FD of that data series.

It should be mentioned that since the data recorded from electrode T4 was noisy almost for all the subjects, we had to remove it in the preprocessing stage and we only had 18 electrodes in our analysis. The cleaned EEG data was also filtered in 12 frequency bands prior to FD calculation including Delta [1-4 Hz], Theta [4-8 Hz] , Alpha I [8-10 Hz], Alpha II [10-12 Hz], Alpha [8-13 Hz], Beta I [12-15 Hz], Beta II [15-18 Hz], Beta III [18-25 Hz], Beta IV [25-30 Hz], Beta [13-30 Hz], lower Gamma [30-40 Hz], and Total band [1-40 Hz].

### Statistical analysis 

In this study, group differences in Katz FD between OCD patients and HCs were statistically evaluated. After checking the normality assumption for the Katz FD, using the Kolmogorov-Smirnov test, a two-sample t-test was applied. A threshold of p<0.05 (Fisher permutation) was considered to distinguish the significant variations of Katz FD in the two groups of participants. 

A 3-way ANOVA was also performed to present the influences of electrodes, frequency bands, and the group on the EEG fractal dimension. 

## Results

**Figure 3:**
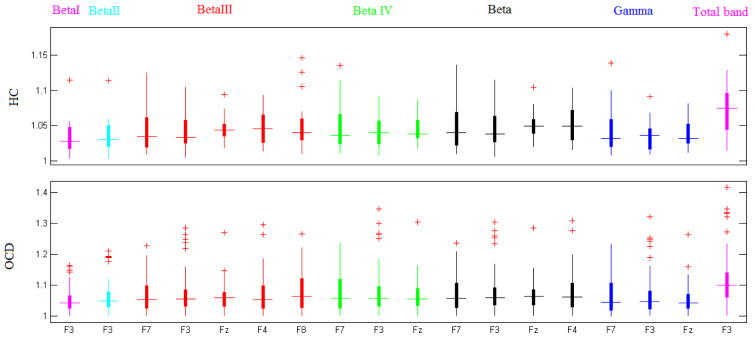
Box plot of HCs' and OCDs' Katz fractal dimension

The fractal dimension of preprocessed EEG signals from 39 OCD patients and 19 healthy controls were separately computed for each frequency band, using Katz's method. Figure 3(Fig. 3) illustrates the boxplots of HCs' and OCDs' band-specific Katz FDs (at channel locations where there are significant differences between the two groups of participants). 

**Figure 4:**
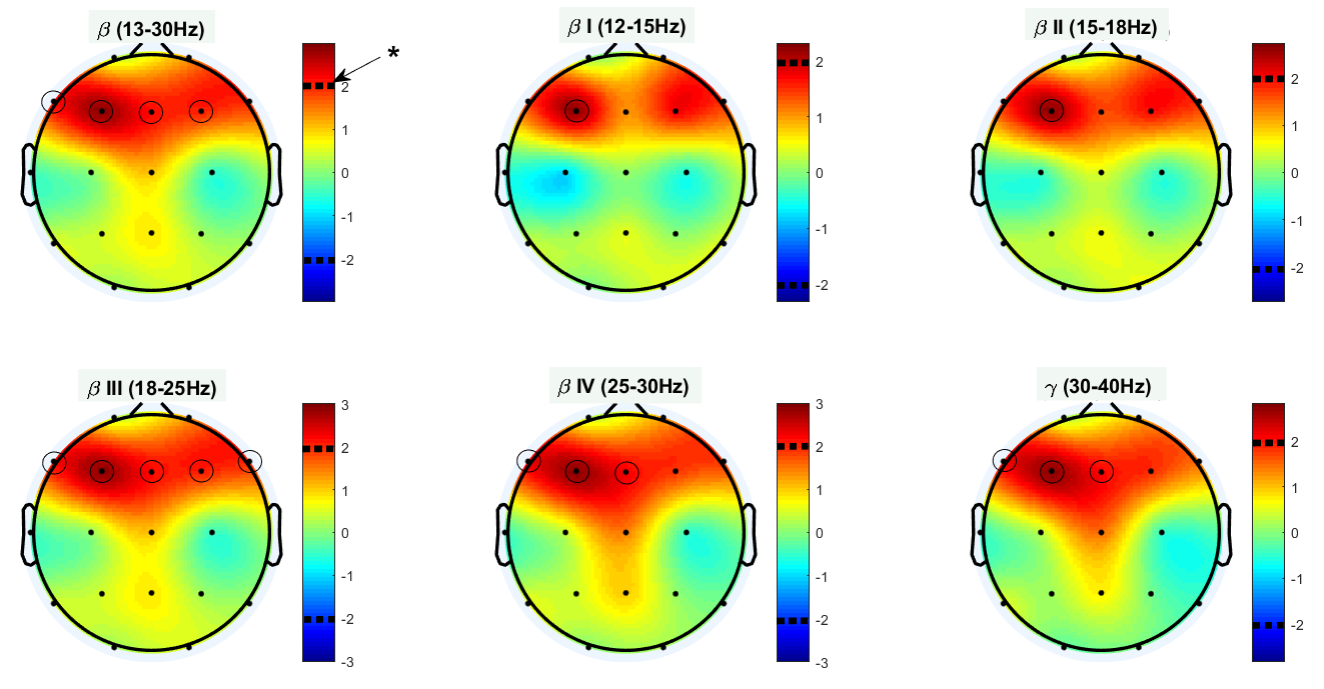
Statistical differences between OCD's and HC's Katz FD in various frequency bands;* denotes p value < 0.05.

Afterward, a two-sample t-test (two-tailed) was performed on the Katz FDs of the two groups. Figure 4(Fig. 4) illustrates topological plots of t-values from statistical comparisons in various frequency bands. The encircled channels are those which show significant differences between the two groups of participants. It could be inferred that the EEG signals from frontal channels (specifically F3) are more complex in high and very high beta band (beta III and beta IV). 

Additional statistical parameters of significant channels (encircled) are reported in Table 2(Tab. 2) in a band-specific manner. These results are consistent with previous findings, and reaffirm the role of frontal regions of the brain in patients with OCD. 

The results of three-way ANOVA, presented in Table 3(Tab. 3), showed that there was no significant influences of electrodes (18 channels), frequency bands (Delta, Theta, Alpha I, Alpha II, Alpha, Beta I, Beta II, Beta III, Beta IV, Beta, lower Gamma, and Total band), and the group (OCD, and HC) on the EEG fractal dimension (F=0.18, df=187, SS=0.0712, p=1). In addition, no significant interaction between electrodes sites and frequency bands on the EEG fractal dimension was observed (F=1.05, df=187, SS=0.4177, p=0.3). Nevertheless, the results showed significant interaction of electrodes sites and groups (F=5.94, df=17, SS=0.2144, p=0.00), as well as influence of interaction of frequency bands and groups (F=2.14, df=11, SS=0.05, p=0.0148) on the EEG fractal dimension. 

### Association of brain complexity and behavioral performance

**Figure 5:**
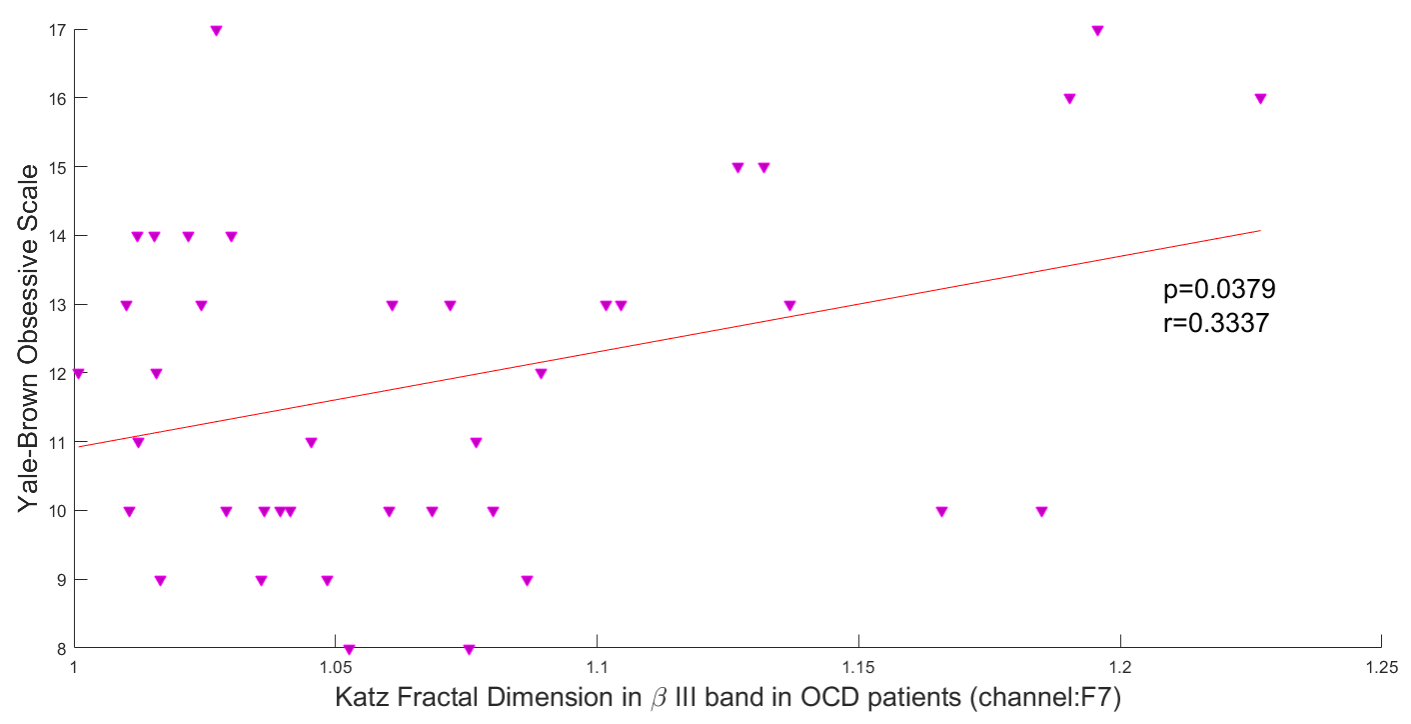
Correlation between obsession scores and Katz FD in high beta frequency band; r denotes Pearson's correlation coefficient, and p denotes the related p value.

**Figure 6:**
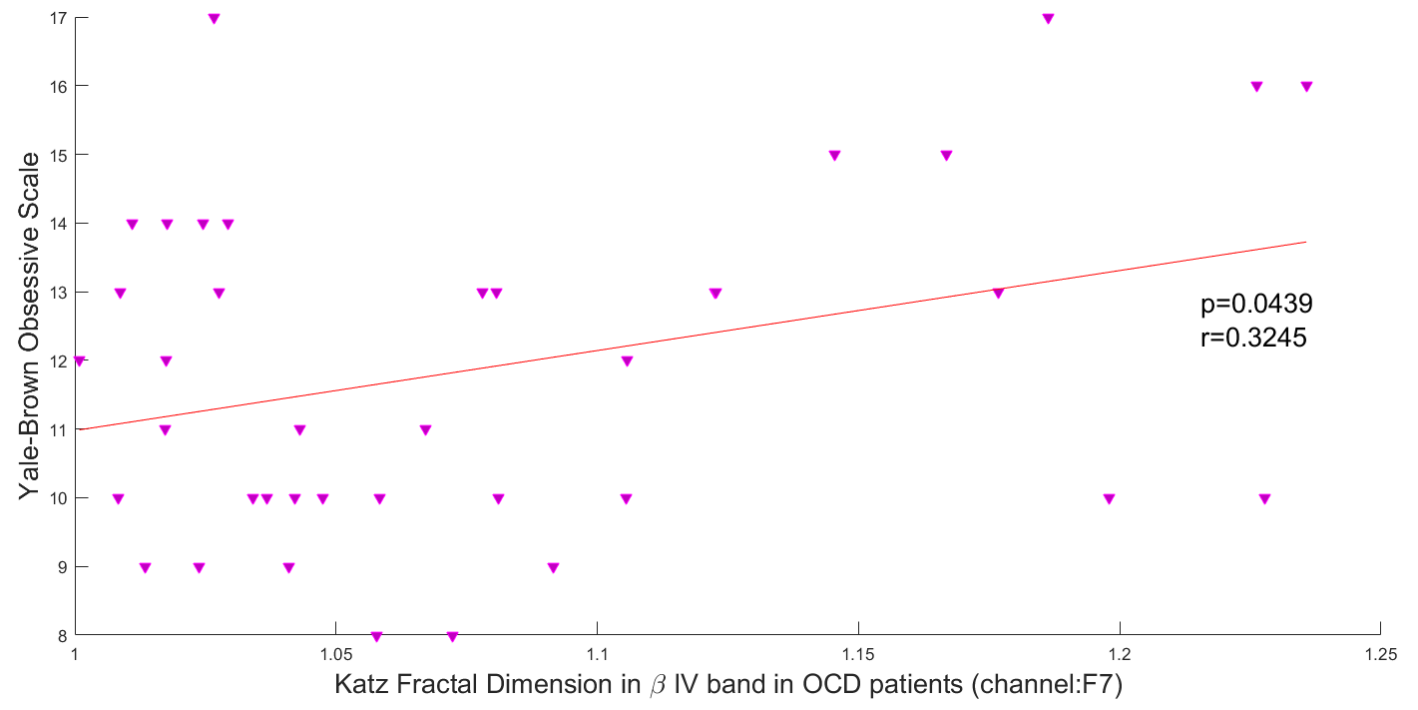
Correlation between obsession scores and Katz FD in very high beta frequency band; r denotes Pearson's correlation coefficient, and p denotes the related p value.

**Figure 7:**
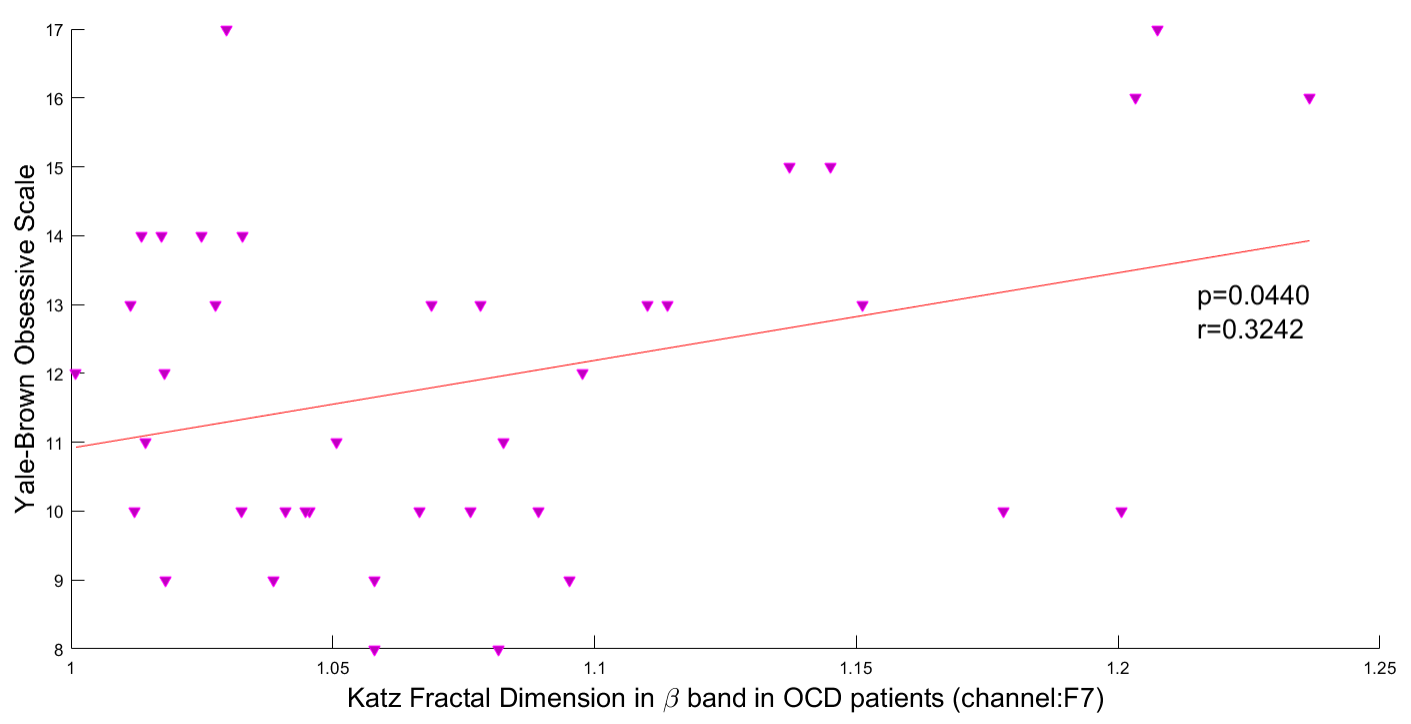
Correlation between obsession scores and Katz FD in beta frequency band; r denotes Pearson's correlation coefficient, and p denotes the related p value.

In this study, the Pearson correlation has been used to discover the strength of correlation between Katz FD and the OCD patients' scores on the Y-BOCS. Results reveal a significant positive correlation between Katz FD and obsession scores on the Y-BOCS in beta III, beta IV, and total beta band of F7 channel. Figures 5(Fig. 5), 6(Fig. 6) and 7(Fig. 7) show the scatter plots of obsession scores versus Katz FDs of EEG signals from OCD patients, along with Pearson correlation coefficients (r). This correlation can be interpreted as the link between behavioral and cognitive dysfunctions (obsession scores), and the structural changes in the brain (Katz FD).

## Discussion

Under the assumption that EEG signals show underlying neural processing, nonlinear analysis of the EEG data could help to study the dynamical properties of information processing, since the underlying neural systems generate EEG potentials (Mölle et al., 1999[36]). In this paper, the complexity of EEG data in OCD individuals and healthy controls was evaluated by calculation of the Katz fractal dimension of EEG data. Our findings highlighted a significant increase of Katz FD in the frontal regions - of the brain in OCDs as compared to HCs. The pattern of changes in FDs was observed in all frequency bands; however, significant changes were only identified in the beta and gamma bands. The most significant difference was observed at the high beta frequency band, and more specifically at the F3 channel location. Since higher values of Katz FD point to higher complexity in the dynamics of EEG, it can be stated that information processing in the frontal regions in OCD is getting more complex as compared to HCs. Previous studies have also pointed to a link between OCD and functions of the frontal brain regions. These results indicate that OCD affects the entire brain in all frequency bands and is not locally restricted, yet it seems to have a stronger influence on the frontal regions at high beta. Previous works denote that the striatum dysfunction influence the implicit learning in the OCD patients (Koen and Stein, 2015[27]) and a repetitive stimulation of projection between orbitofrontal cortex and ventral striatum might be used to compensate this failure. Therefore, origin of obsessions in OCD can be traced back to the fronto-striato-thalamic circuit (cortico-basal ganglia-thalamo-cortical loop's) inability to restrict implicitly processed information (Koen and Stein, 2015[27]). This inability could cause the information to be processed in explicit information processing systems. This description of obsessions is consistent with our finding that higher Katz FD is connected to exaggerated information processing in OCD patients. For instance, an exaggerated fixation on unimportant cues might increase the involvement of the frontal regions and cause failure to filter out extraneous information in OCD individuals (Antony and Stein, 2008[6]).

Additionally, dysfunction of the frontal cortex could also compromise the inhibitory mechanism (Garcia-Junco-Clemente et al., 2017[16]), which is a major indication of OCD (Chamberlain et al., 2005[13]). Moreover, the decision making process is also impaired in OCD (Abramovitch and Cooperman, 2015[2]; Aydın et al., 2014[9]), which is also related to the role of frontal regions and striatum. An increased activity is generally seen in the left frontal region and is presumed to be asymmetric (Grützmann et al., 2017[20]). Although findings on the dysfunctions of the frontal cortex in OCD patients are varying (Gonçalves et al., 2016[17]; Lewin et al., 2014[30]; Nakao et al., 2014[37]; van den Heuvel et al., 2005[46]; Wong et al., 2015[48]), a rather fair increase of the beta band activity has been observed in numerous studies (Purcell et al., 1998[41]; Rubia et al., 2011[43]). As a result, we believe that exaggerated information processing in the frontal regions of the brain would increase the complexity of information processing in this region in OCD patients.

The particular distribution of Katz FD along frontal and temporal regions in OCD patients can be used as a potential neuro-marker as a means to better understand OCD and to provide patient-tailored treatments. A recent study points to a hyper-connectivity between frontal and temporal brain regions in OCD patients, representing complex processing of information in these areas (Yazdi-Ravandi et al., 2018[50]). However, the in-detail specifics of variations of the dynamic complexity of information processing in these regions were not discussed. Here, we further scrutinized how the complexity of the aforementioned information processing changes in the frontal and temporal regions. We hope that our findings help to improve the theoretical understanding of brain function in OCD and encourage future research on the inner-workings of OCD. 

As limitations of this study, by considering the ethical issues, we only recruited the OCD patients under medication in our study. However, the medication could influence the functionality of the brain. Therefore, the results may not be directly extended to all OCD patients. Moreover, since the EEG captures cortical activities, we have only discussed cortical regions in our findings, while according to the cortico-basal ganglia-thalamo-cortical loop model, other areas are involved as well. In terms of data analysis, recording with higher number of channel will also provide the opportunity to combine individual electrodes into electrode clusters and reduce the number of individual tests.

## Conclusion

In this study we presented how complexity of information processing at the frontal regions is increased in OCD patients. The pattern of increase mainly was observed at beta and gamma bands and more significantly at the higher beta band frequencies. Interestingly, the significant changes of EEG FDs were positively correlated with the severity of obsessions. These results indicate that repetitive activations of the orbitofrontal cortex might be a compensatory mechanism for the ventral striatum dysfunction in OCD patients. 

## Notes

Ali Ghaleiha and Reza Khosrowabadi (Institute for Cognitive and Brain Sciences, Shahid Beheshti University, Evin Sq., Tehran 19839-63113, Iran; Tel: +98(0)9101738501, Fax: +98(21)22431998, Email: r_khosroabadi@sbu.ac.ir) contributed equally as corresponding authors.

## Acknowledgements

This paper is part of a Ph.D. thesis supported by Hamadan University of Medical Sciences (Grant No: 94011854). The authors gratefully acknowledge the financial support provided by the vice chancellor of research and technology of Hamadan University of Medical Sciences.

## Figures and Tables

**Table 1:**
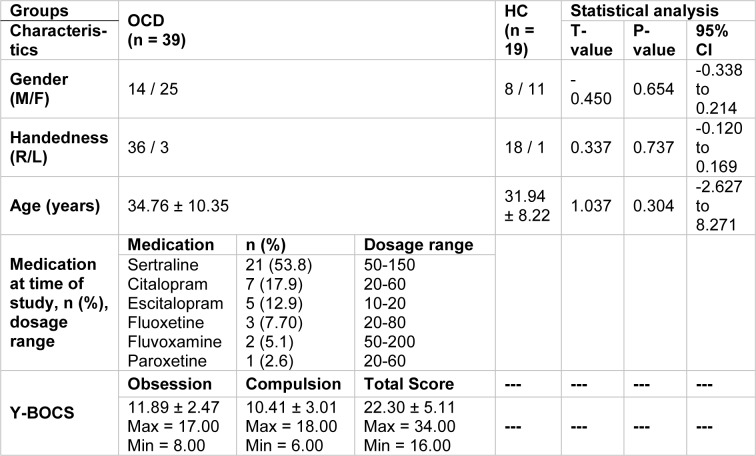
Demographic characteristics of the participants

**Table 2:**
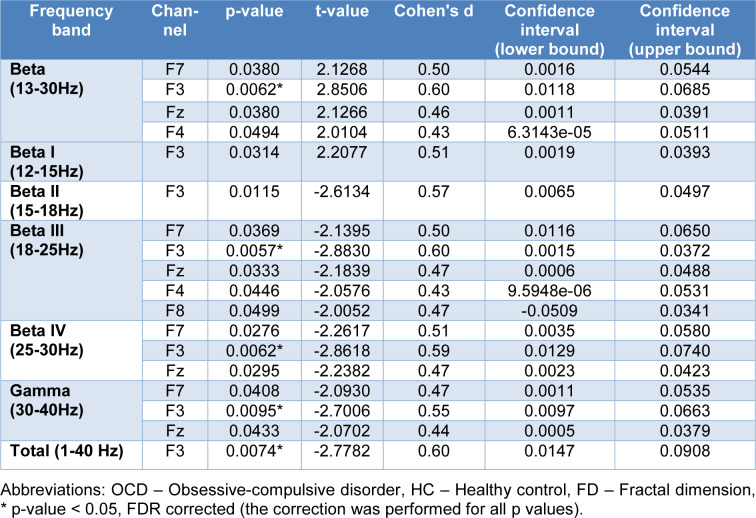
Significant statistical differences in EEG FDs between OCD patients and HCs

**Table 3:**
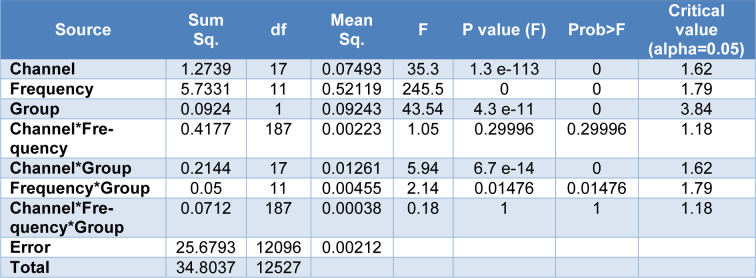
The influences of electrodes, frequency bands, and the group on the fractal dimension using a 3-way ANOVA
